# Influence of the Numbers of Players in the Heart Rate Responses of Youth Soccer Players Within 2 vs. 2, 3 vs. 3 and 4 vs. 4 Small-sided Games

**DOI:** 10.2478/v10078-011-0027-8

**Published:** 2011-07-04

**Authors:** A Dellal, R Jannault, M Lopez-Segovia, V Pialoux

**Affiliations:** 1 Department of Sports Science and Exercise, Strasbourg University, France; 2 Tunisian Research Laboratory “Sport Performance Optimisation” – Nat. Centre of Med. and Sci. in Sport -El Menzah, Tunisia.; 3 Olympique Lyonnais FC (soccer), Lyon, France; 4 Université Claude Bernard Lyon 1, Centre de Recherche et d’Innovation sur le Sport, 69622 Villeurbanne Cedex, France.; 5 Research Section Soccer Federation of the Region of Murcia, Murcia, Spain

**Keywords:** youth soccer players, physiological responses, reserve heart rate, fitness training, aerobic training

## Abstract

The purpose of this study was to compare heart rate (HR) responses within and between small-sided games (SSG) training methods in elite young soccer players. Twenty-seven youth soccer players (age: 16.5 ± 0.5 years, height: 174.5 ± 5.5 cm, weight: 62.9 ± 8.3, velocity at maximal aerobic speed (MAS): 15.9 ± 0.9 km.h^−1^) performed 3 different SSG (2 vs. 2, 3 vs. 3, 4 vs. 4 without goalkeeper). In each SSG, HR was continuously measured and expressed as a mean percentage of HR reserve (%HRreserve). The mean %HRreserve calculated during the SSG was significantly lower during 4 vs. 4 (70.6 ± 5.9 %) compared to 2 vs. 2 (80.1 ± 3.6 %, p<0.001) and 3 vs. 3 (81.5 ± 4.3 %, p<0.001) SSG. Regardless of the time spent above 60, 65, 70, 75, 80, 85 and 90 % of HRreserve, 4 vs. 4 solicited lower percentage of time than 3 vs. 3 and 2 vs. 2. Intersubject coefficients of variation were significantly higher during 4 vs. 4 compared to 2 vs.2 and 3 vs. 3. The %HRreserve after 30s of recovery was significantly higher for 3 vs. 3 (70.6 ± 5.3 %) compared to 2 vs. 2 (65.2 ± 4.8 %, p<0.05) and 4 vs. 4 (61.6 ± 9.3 %, p<0.05). In conclusion, this study demonstrates that the physiological demands is higher during 2 vs. 2 and 3 vs. 3 compared to 4 vs. 4 in youth soccer players. This difference could be due to that young soccer players do not have the same technical ability and experience as adult players and thus, their activity during the 2 vs. 2 and 3 vs. 3 induces a greater physical demand due to their lack of experience. The age of the players could be linked with the physical demands within small-sided games.

## Introduction

Physiologically, youth soccer players present lower values of maximal oxygen uptake (VO_2_max) than adults (Stolen et al., 2005). Maximal aerobic capacity is considered a key factor in modern soccer. Indeed, a high value of VO_2_max allows one to cover a large total running distance (McMillan et al., 2005), improve the ability to repeat sprints, and perform high-intensity actions both with and without the ball (Durandt et al., 2006; Hoff et al., 2002). Moreover, Helgerud et al. (2001) have shown that the technical activity and total sprinted distance during the match-play of youth soccer players as improved with the increase in VO_2_max. Consequently, it appears essential for the coaches to pay attention to the development and the optimization of aerobic capacity in youth players.

In this context, intermittent exercises are regularly included in soccer training because it was described that this method solicits a high proportion of VO_2_max and improves maximal aerobic capacity both in adult (Dellal et al., 2010) and in youth soccer players (Wong et al., 2009). However, recent data drawn from the Spanish and English first league suggest that training methods should integrate ball possession (Dellal et al., 2011b). Indeed, the total duration of the individual ball possession was ranged from 41.7 to 84.4 seconds per match-play while the number of ball contact per possession ranged from 1.74 to 2.24 (Dellal et al., 2011b). Moreover, 1.2% to 2.4% of the total distance is covered with the ball (Di Salvo et al., 2007).

Therefore, small-sided games (SSG) are commonly used within training in order to simultaneously solicit the tactical, technical, and physical components (Rampinini et al., 2007). Williams and Frank (1998) suggested the SSG as a useful tool allowing the identification of talent in youth soccer players. Coaches favor the use of SSG in order to increase the number of ball contacts and the total distance covered in sprinting, and to confront the players in a greater technical difficulty than those found during a match-play (Dellal et al., 2011a). Previous studies have reported that the rules, game formats and duration, pitch ratio per player (pitch area divided per number of players) and pitch dimension influence the technical and physical demands within SSG (Kelly and Drust, 2009; Hill-Haas et al., 2010; Rampinini et al., 2007; Tessitore et al., 2006). Although the 2 vs. 2, 3 vs. 3 and 4 vs. 4 are presented as the most SSG used in elite soccer, to the best of our knowledge, physiological responses within these three specifics SSG in youth soccer players was not yet investigated. Specifically, heart rate (HR) responses in youth soccer players were examined during SSG (from 2 vs. 2 to 6 vs. 6) (Hill-Haas et al., 2009a; Hill-Haas et al., 2009b; Katis and Kellis, 2009; Platt et al., 2001). Although the 2 vs. 2, 3 vs. 3 and 4 vs. 4 are presented as the most SSG used in elite soccer, some methodological issues emerging from these previous studies do not allow to provide a clear statement regarding the effects of the numbers of players in SSG. First, the inconsistency of rules and the game format between the studies may induce bias in the interpretation of results. Secondly, the HR responses were usually expressed as percentage of maximal HR (%HRmax) (Hill-Haas et al., 2009b; Hill-Haas et al., 2009a; Hill-Haas et al., 2009b; Katis and Kellis, 2009; Platt et al., 2001; Kelly and Drust, 2009; Platt et al., 2001; Rampinini et al., 2007) and, contrary to the percentage of the reserve HR (%HRreserve), this HR expression does not take into account the individual resting HR which can lead to an approximation of the intensity and the inter-individual comparison is not efficient.

Consequently, the aim of the current investigation was to examine the influence of the modulation of the number of players in the three most popular SSG used in soccer (i.e. 2 vs. 2, 3 vs. 3 and 4 vs. 4) on the HR responses expressed in %HRreserve and on the blood lactate in elite youth soccer players. It is hypothesized that the number of players induces significantly different physiological responses with almost different intersubject coefficient of variation. Secondly, it was hypothesized that the time spent at a high proportion of their %HRreserve and consequently of their VO_2_max, varied between these SSG.

## Material and methods

### Subjects

Twenty-seven elite soccer outfield players participated in this study (age: 16.5 ± 0.5 years, height: 174.5 ± 5.5 cm, weight: 62.9 ± 8.3 kg). The subjects played in the U17 French national first division and trained 8 hours (3 technical-tactical training sessions and 2 physical training sessions), plus one official game per week. More specifically, during the first part of the competitive training season, training sessions were placed on Tuesday and Thursday. After a development of aerobic capacity and strength during the pre-competitive part of the season, the development of maximal aerobic speed via SSGs and interval-training sessions was usually conducted on Tuesday while development of maximal speed and explosive power of lower limbs was performed on Thursday. Subjects had a soccer experience of 8.1 ± 2.5 years with at least 2 years of national level competitions. The experiment was performed during the second part of the competitive season (between last week of February and last week of March).

The study was conducted according to the ethical standards in sport and exercise science research (Harriss and Atkinson, 2009). The local university ethics committee approved the protocol. All subjects received a detailed explanation about the aims of the study. Appropriate informed parental consent has been gained for all subjects.

### Experimental Approach of the Problem

All the 27 subjects performed a maximal aerobic speed (MAS) test and then 24 of them were submitted to 3 different SSG on a natural grass soccer field. The MAS test and the 3 SSG were all performed 3 days after an official game (i.e. Wednesday) during the same hours of the day (between 5:00 and 6:30 PM) and separated at least by one week. An appropriate standardized warm-up (general physical preparation with articular and muscular mobilization) was performed before each SSG. Hydration was *ad libitum* during each testing sessions.

### Procedures

#### Maximal aerobic speed test

Maximal aerobic speed (MAS) was assessed using the VAMEVAL test described by Cazorla and Léger (1993), which allows to provide the smallest velocity associated with VO_2_max (*v*VO_2_max). Briefly, the test starts at a running speed of 8 km.h^−1^ and increases by 0.5 km.h^−1^ each minute until exhaustion. The subjects adjusted their running speed to auditory signals at 20-m intervals, delineated by visual marks along a 200-m long oval drawn on the soccer field. This test estimated the subjects’ maximal aerobic speed. The MAS is the speed in km.h^−1^ of the last 1-minute stage completed by the subject. The uncompleted 1-minute stages were not taken into account.

#### Small-sided games (SSG)

From the 27 subjects tested for MAS, 24 were evaluated over the 3 SSG situations (no goalkeeper, no goal): 2 vs. 2 (8 periods of 2 min separated by 1 min of passive recovery, with a pitch of 20 m × 25 m), 3 vs. 3 (6 periods of 3 min separated by 1 min 30 s of passive recovery, with a pitch of 25 m × 30 m) and 4 vs. 4 (4 periods of 4 min separated by 2 min of passive recovery, with a pitch of 28 m × 35 m). The pitch ratio per players (pitch area divided per the number of players) was similar with respectively 125 m^2^ for the 2 vs. 2 and the 3 vs. 3, while 122.5 m^2^ for the 4 vs. 4. Game duration was based on coaching experience. The SSG were played according to the “stop-ball” rules. All the players of the team had to be located in the offensive side of pitch to validate the point. Each SSG were performed with 2 coaches around the perimeter of pitch in order to encourage the players and to provide new balls when necessary to allow continuity of play during the matches.

#### Heart Rate Measurements (HR)

HR was recorded in 1-second intervals during the MAS test and the 3 SSG using HR monitors (Polar RS800, Polar Electro, Finland) and analysed with Polar Pro Trainer 5 software (Polar Electro, Finland). Resting HR was determined as the lowest HR measured during a 10 min supine position before each SSG testing. Maximal HR (HRmax) was determined as the highest average values over 5 s during the MAS test. HRreserve was then calculated as the difference between HRmax and resting HR registered. The percentage of HRreserve (%HRreserve) was calculated by the following formula (Karvonen et al., 1957) previously used by Dellal et al. (2008): %HRreserve = (exercise mean HR - resting HR) / (HRmax - resting HR) x 100. During the SSG, the mean %HRreserve, the percentage of time spent above 60, 65, 70, 75, 80, 85, 90 and 95% of HRreserve were calculated. In addition the %HRreserve measured 30 s after the end of each SSG as an indicator of post-exercise cardiac recovery.

#### Post-Exercise Blood Lactate [LA]

The measurement of blood lactate concentration was performed to know the impact of each SSG on anaerobic metabolism. Indeed, small-sided games can be characterized by repetitions of runs at supra-maximal intensity. In this context, anaerobic metabolism was involved in the energy contribution of SSGs inducing an increase in blood lactate concentration. Blood lactate concentration was measured with the Lactate Pro device (Arkray Inc, USA) 3 minutes after the end of each SSG. This device has been shown to provide valid indications of blood lactate concentrations (Pyne et al., 2000). The use of this portable analyzer on the field presented a high correlation (r=0.99) with the values found in a laboratory, and consequently, this analyzer can be used interchangeably within the field and laboratory in competitive sport (McNaughton et al., 2001).

### Statistical analysis

All results are reported as means and standard deviations (means ± SD). Intersubject coefficient of variation (CV) was calculated for each percentage time selected (60, 65, 70, 75, 80, 85, 90, and 95 %) of HRreserve for all the 2 vs. 2, 3 vs. 3 and 4 vs. 4. After confirming normal distribution with a Kolmogorov-Smirnov test, the difference between the 3 SSG (time effect) for each described variables were analysed using a one-way repeated measures ANOVA followed by Tukey post hoc test. Statistical analyses were performed with analytical software (Version 15.0, SPSS, Chicago, IL, USA). Differences were considered significant at p<0.05.

## Results

**Figure 1:**
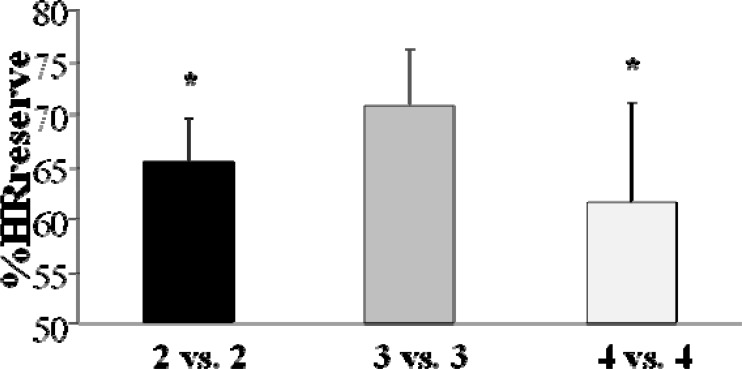
Effect of 2 vs. 2, 3 vs. 3 and 4 vs. 4 small-sided games on the mean percentage of heart rate reserve (%HRreserve) calculated on the total and effective play time. *p<0.05 and **p<0.01 compared to 4 vs. 4

The mean %HRreserve calculated during the total duration (exercise and recovery periods) of the 4 vs. 4 SSG was significantly lower (70.6 ± 5.9 %) as compared to the 2 vs. 2 (80.1 ± 3.6 %, p<0.001) and the 3 vs. 3 SSG (81.5 ± 4.3 %, p<0.001) (Figure 1). Similarly, the %HRreserve calculated during the effective time of the SSG was significantly lower during 4 vs. 4 (78.4 ± 5.8 %) compared to 2 vs. 2 (85.1 ± 4.3 %, p<0.05) and 3 vs. 3 (87.8 ± 4.1 %, p<0.01) (Figure 1).

**Figure 2:**
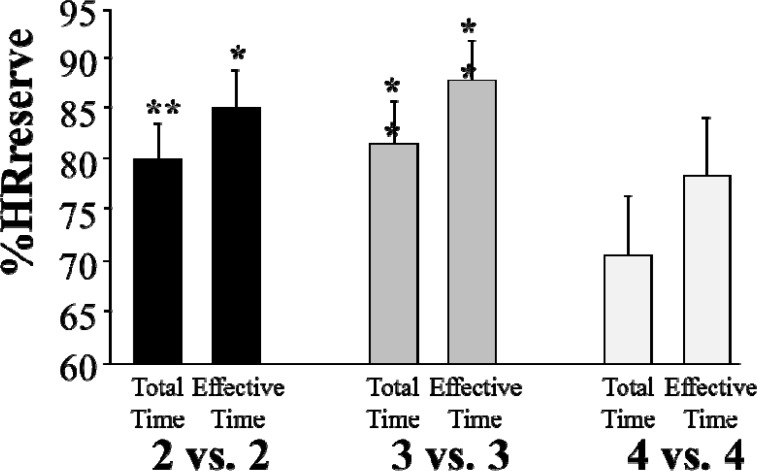
Percentage of heart rate reserve (%HRreserve) measured after 30s of recovery of 2 vs. 2, 3 vs. 3 and 4 vs. 4 small-sided games. *p<0.05 compared to 4 vs. 4.

The percentages of time spent above 60, 65, 70, 75, 80, 85, 90 and 95% of HRreserve are detailed in Table 1. Excepted over 95% of HRreserve, the percentage of time spent over all the pre-determined %HRreserve was significantly higher for 3 vs. 3 compared to 4 vs. 4. For 60, 65, 70, 75, 80 and 85% of HRreserve, the percentage of time was significantly higher for 2 vs. 2 compared to 4 vs. 4. Results show no significant difference in the percentage of time spent over any of the predetermined %HRreserve between 2 vs. 2 and 3 vs. 3. Intersubject CVs were significantly higher during 4 vs. 4 compared to 2 vs. 2 and 3 vs. 3 (Table 2). The percentage of HRreserve after 30s of recovery post-exercise was significantly higher for 3 vs. 3 (70.6 ± 5.3 %) as compared to the 2 vs. 2 (65.2 ± 4.8 %, p<0.05) and 4 vs. 4 (61.6 ± 9.3 %, p<0.05) (Figure 2). The blood lactate measured 3 minutes after each SSG were not significantly different (2 vs. 2: 6.32 ± 2.35 mmol.l^−1^; 3 vs. 3: 7.48 ± 1.73 mmol.l^−1^ and 4 vs. 4: 7.07 ± 4.36 mmol.l^−1^).

## Discussion

The main objective of this study was to investigate the differences in physiological responses (HR responses and [LA]) in youth soccer players within various SSG formats (2 vs. 2, 3 vs. 3 and 4 vs. 4) with similar pitch ratio per player. The main finding was that the total time spent at high proportion of the %HRreserve, thus probably of the VO_2_max, was greater within the 2 vs. 2 and the 3 vs. 3 SSG compared to the 4 vs. 4 despite the fact that the reached values of HR were similar during the different SSG. Furthermore, no difference was observed concerning both the percentage of the decrease of the HR post-effort and [LA] concentration throughout all the SSG examined.

Many of the previous studies examining the HR responses during SSG used the HR expressed in percentage of maximal HR (%HRmax). This method of analysis may result in false calculated intensity as it has been showed that the %HRreserve is a better indicator and tool to compare the inter-individual HR responses (Dellal et al., 2008; Hoff et al., 2002). The different SSG examined in the present study allows one to reach the same values of %HRreserve than those previously described (e.g. Kelly and Drust, 2009). The time spent at a high proportion of the %HRreserve was significantly lower within the 4 vs. 4 (p<0.001) both expressed in function of the total duration or the effective duration of the SSG, confirming data of Katis and Kellis (2009) which reported a lower number of players within the SSG induced greater HR responses. Moreover, the HR responses presented a greater heterogeneity (intersubject CV) throughout the 4 vs. 4 suggesting that the parameters of this SSG do not solicit high level of aerobic metabolism in some of the players. Therefore, it could be hypothesized that the training including 2 vs. 2 and the 3 vs. 3 SSG has a better effect on maximal aerobic capacity and VO_2_max as compared to a 4 vs. 4 in youth players. The improvement of VO_2_max requires intense HR responses but also a large time spent at high percentage of VO_2_max (Dupont and Berthoin, 2004).

In this context, coaches favored the use of the short duration intermittent exercise. In adult soccer, SSG had been considered as a useful tool allowing the optimization of VO_2_max similar to the high-intensity intermittent exercises (Dellal et al., 2010). The results of the present study suggest that the 2 vs. 2 and 3 vs. 3 SSG could also stimulate a high percentage of VO_2_max in youth soccer players. Youth soccer players need to develop their physical performance (maximal aerobic capacity and ability to repeated sprints), technical, and tactical abilities. Therefore, the use of 2 vs. 2 and 3 vs. 3 SSG seems to represent an ideal alternative to optimize simultaneously these components in youth soccer players.

The technical and the physical activities of the players during the SSG is less homogenous when the number of players is increased (Hill-Haas et al., 2009b; Di Salvo et al., 2007). The results of the present study confirmed this finding in showing greater heterogeneous physiological responses in the 4 vs. 4 as compared to the 2 vs. 2 and the 3 vs. 3, regarding the HR responses and the blood lactate concentration. Therefore, these differences could be linked to physical, technical, and tactical demands within the different SSG. The 2 vs. 2 and the 3 vs. 3 was described as SSG induced more duels, percentage of total distance covered at high intensity, directional changes, dribbling or ball contacts as compared to SSG with greater number of players (Della et al., 2011a; Hill-Haas et al., 2010; Katis and Kellis, 2009; McMillan et al., 2005). Although, the pitch ratio per players was similar for all the SSG analysed, the number of players influences the technical difficulty and the high-intensity actions. Indeed, during the 2 vs. 2 and 3 vs. 3, the players are always concentrated on the play and have to be continuously moving in order to create spaces by the means of turns, direction changes or sprints. These high-intensity actions are suggested to be linked to the greater technical difficulty combined to the possible lower duration to perform particular technical actions. The players have to perform the offensive actions (passes, dribbling and strikes at the goal) and the defensive actions (tackling and pressure on the players who have the ball) more quickly and at a greater frequency. These requirements of bout and execution speed in SSG imply the combination of physical, perceptible, technical and tactical factors. Youth soccer players need to develop their physical performance, but also their technical, perceptible, and tactical abilities, and by the findings obtained in the present study 2 vs. 2 and 3 vs. 3 SSG seems to represent an ideal alternative to optimize the training process and could be used to develop simultaneously these components in youth soccer players. When the SSG include goals, Katis and Kellis (2009) and Platt et al. (2001) showed that players performed more short passes, tackling, shooting and dribbling during a 3 vs. 3 as compared to a 5 vs. 5 and a 6 vs. 6 SSG. The psychological and physiological stresses on the players are increased when the number of players is decreased, especially when their team is in ball possession. The modern high-level soccer requires playing quickly in offensive situations and to put pressure on the opponent as quickly as possible in defensive situations (Dellal et al., 2011b).

In this context, the SSG solicit all the elements considered as the key factors in modern soccer (Lago-Penas et al., 2010). Therefore, coaches have to favor the SSG in their training sessions in order to recreate the high-intensity actions found in a match-play. Furthermore, according to Williams and Frank (1998), SSG have to be considered a useful tool allowing the identification of talent in youth soccer players, especially the 2 vs. 2 and 3 vs. 3. However, coaches have to pay attention to the fact that youth soccer players do not present the same technical ability as the adult or elite players. The youth soccer players presented greater anthropometrics and physiological differences according to their positions than those observed in adult players (Wong et al., 2010). The number of periods and their duration has to be precisely determined. Moreover, Dellal et al. (2011a) observed that the number of ball contacts per individual possessions influenced technical activities, physical performance and physiological responses within the SSG. Therefore, according to the playing-level and the characteristics of the young players, the coaches have to choose between a free play rule and a controlled number of ball contacts authorized per possession. It is also suggested that the game rules have to be varied in order to create different adaptation in youth soccer players.

To conclude, the present study reveals that the small-sided games (SSG) might induce an HR response sufficient to stimulate maximal oxygen uptake (VO_2_max) in youth soccer players. The time spent at a high proportion of the percentage of the reserve HR (>90% of the HRres) was greater when the SSG was played with fewer players (i.e. 2 vs. 2 and 3 vs. 3). Moreover, the homogeneity of the physiological responses (blood lactate concentration and HR responses) of the players was lower within the 4 vs. 4 SSG compared to the 2 vs. 2 and the 3 vs. 3, probably due to a greater influence of the tactical component. Finally, all these elements could help in planning of seasonal programs and multifunctional aspects of specific training sessions in youth soccer players.

## Figures and Tables

**Table 1:** Percentage of time spent over selected percentage of reserve heart rate (%HRreserve) during 2 vs. 2, 3 vs. 3 and 4 vs. 4 small-sided games.

**%HRreserve**	**> 60**	**> 65**	**> 70**	**> 75**	**> 80**	**> 85**	**> 90**	**> 95**
**2 vs. 2**	93.9 ± 5.7^**^	88.5 ± 7.1^**^	80.6 ± 7.1^**^	72.1 ± 8.0^**^	61.0 ± 11.9^**^	41.8 ± 19.5^**^	19.3 ± 17.3^**^	4.3 ± 6.9
**3 vs. 3**	94.2 ± 6.2^**^	89.1 ± 6.8^**^	82.9 ± 7.2^**^	75.6 ± 7.9^**^	66.5 ± 11.6^**^	50.1 ± 19.6^**^	31.6 ± 20.9^**^	12.0 ± 13.7
**4 vs. 4**	73.6 ± 8.6	72.9 ± 11.1	65.9 ± 13.5	55.0 ± 16.8	41.6 ± 20.2	20.5 ± 19.7	7.2 ± 12.2	1.9 ± 5.1

** p<0.001 and

* p<0.05 compared to 4 vs. 4.

**Table 2:** Inter-subject coefficient of variation (CV) of the percentage of time spent over selected percentage of reserve heart rate (%HRreserve) during 2 vs. 2, 3 vs. 3 and 4 vs. 4 small-sided games.

**%HRreserve**	**> 60**	**> 65**	**> 70**	**> 75**	**> 80**	**> 85**	**> 90**	**> 95**
**2 vs. 2**	6.6	7.6	8.7	10.4	17.5	38,4	66.2	114.6
**3 vs. 3**	6.0	8.0	8.8	11.1	19.5	46,7	89.7^*^	160.8^*^
**4 vs. 4**	10.9^*^	15.2^**^	20.5^**^	30.6^**^	48.5^**^	91.6^**^	169.0^**^	269.2^**^

* p<0.001 and

* p<0.05 compared to 2 vs. 2.
